# Cases of Surgery

**Published:** 1845-10

**Authors:** Daniel Brainard

**Affiliations:** Professor of Surgery in Rush Medical College, Chicago


### ILLINOIS

### MEDICAL & SURGICAL JOURNAL.

VOL. II. OCTOBER, 1845. NO. 7.

*Cases of Surgery.* By Daniel Brainard, M.D., Professor of Surgery in Rush Medical College.

CASE I.

*Injury of the Head without Fracture of the Skull—Compression of the Brain from Effusion of Blood—Trephining—Recovery.*— H. H., aged 23 years, a robust and athletic man, received several blows with a stick upon the head, in an affray upon the race course, nearly three miles from Chicago, Monday, Sept. 1st., 1845, by which he was stunned at first, but soon recovered. He walked about for some time, then mounted a horse to ride to Chicago, and proceeded about a mile without feeling uneasiness. He then complained of dullness of hearing, and soon after found it difficult to manage his horse, which it became necessary to lead. On arriving at the stable, he lay down, and was soon after found by his companion asleep on the floor. Being roused, he walked a short distance to a covered wagon, in which he slept for the night, and where he was found the next morning in a state of profound coma, from which it was impossible entirely to rouse him.

Medical aid being called, it was observed that an entire loss of motion and a partial loss of sensation existed on the right side of the body, affecting the limbs also, while those of the left were readily withdrawn if smartly pinched. The respiration was deep, but not stertorous; the pulse slow, but full; surface cool. He could with difficulty be made to answer the simplest question. Free blood-letting and other suitable remedies were immediately resorted to, without benefit, however, and on Wednesday, Sept. 3, we were called to see him, and found him in the state above described. The head having been shaven, it was found that no mark of injury existed upon it, except one, very slight, just at the superior angle of the os occipitis, nor could any information be gained as to the precise point upon which the violence was inflicted. The symptoms and the history of the case, left no doubt of the character of the injury and the urgent necessity of an operation, while they afforded no clue to the precise situation of the effusion, or the point upon which the trephin should be applied. In judging of this, two circumstances were taken particularly into account: the first was, the members paralyzed; the second, the point at which effusion most readily takes place. Repeated observation has shown us that when the members of one side are paralyzed from an injury, it is when this has been inflicted upon the central or superior parts of the opposite hemisphere of the cerebrum. It is known also, that extensive effusions of blood from such causes, most frequently take place from the middle meningeal artery, or some of its principal branches. Guided by these facts, we determined on trephining an inch above the left ear, and on a line a little anterior to it. For this purpose, a triangular flap was raised, and some ecchymosis was discovered among the fibres of the temporal muscle. On removing the pericranium, no blood was observed to exude on the surface of the bone, and on removing a circle of this with the trephine, a coagulum instantly protruded. Pieces of this were removed with the finger, until several ounces had come away. The thickness of the coagulum at the point of the opening was not less than an inch, and its limits could not be ascertained. A tent was placed in the opening, and simple dressings applied.

During the operation, the patient suffered considerably, crying out, and requiring to be held, and after the removal of the blood, he manifested consciousness, and moved the members of the right side to a considerable extent. No unfavorable symptoms occurred; his recovery was rapid; and in less than two weeks he was able to start for his own home, in the central part of the State.

To Drs. Maxwell and Herrick, of this city, my acknowledgments are due for their advice and assistance in this case.

CASE II.

*Bony Concretion in the Knee Joint—Extraction by the Subcutaneous Method—Recovery.*—W. G., aged 26 years, of Du Page county, Illinois, visited Chicago for the purpose of obtaining relief for a troublesome affection of the right knee.

*History.* About ten years since, he was attacked with an inflammation of the joint, called rheumatism, which returned at irregular intervals, particularly when much exercise was taken, until the present time. About six months since, he noticed a hard substance, movable in the joint, which at times disappeared beneath the patella. For some months, the attacks of inflammation had been more frequent and severe, so as to entirely preclude him from following any active or laborious employment.

*Present State.* On examination, the articulation was found to contain considerable fluid within the capsule, but there was little tenderness, and no marks of inflammation. The hard body could occasionally be felt on the inside of the patella, but slight pressure caused it to disappear.

*Extraction.* It being impossible to retain the substance in a fixed position, by the aid of a bandage or knee-cap, it was determined to extract it; and the operation was performed, with the assistance of Drs. Herrick and Blaney, Sept. 7, 1845, in the following manner: The patient being placed upon his back, and the limb extended, the body was fixed by pressure with the fingers upon the anterior surface of the inner condyle of the femur. An incision, over an inch in length, and an inch within the situation of the substance, was then made through the integuments. A bistoury being carried beneath these, an opening was made through the synovial capsule, directly upon the movable body. This was then extracted by the aid of the tenaculum and forceps, a work of some difficulty on account of its extreme hardness, which did not admit of its being penetrated in the slightest degree. The wound was then brought together by stitches and adhesive straps, evaporating lotions applied, and the member kept in a state of perfect repose. The body extracted was about twice the size of a large bean, spherical, smooth, and encrusted with cartilage upon its surface; hard and osseous within. At the end of a week, the wound was quite closed, and the patient enabled to walk about as usual, but by an injudicious attempt to travel, considerable inflammation was excited, which required blood-letting and other reducing means to subdue it, thus adding another to the numerous proofs already on record, of the harmless nature of such injuries, when perfect immobility of the member is preserved, and of their serious character when this is neglected.

CASE III.

*Foreign body in the Bronchial Tubes—Laryngo-Tracheotomy—Death—Post Mortem appearances.*—This case was of a child a little over twelve months old, which, on the 2d of September, 1845, while swallowing a piece of water melon containing seeds, (which he had seized,) was attacked with a violent paroxysm of coughing, amounting almost to suffocation, when this had subsided, a sound, called by the mother "purring," was heard. The accessions of coughing were frequent and violent from that time until the 19th of September, when the child was brought to us for examination.

*Present State.* The *en-bon-point* of the infant was good, respiration frequent, considerable blueness of the veins which ramify along the head, and a distinct murmur or "purring" sound was heard during the inspirations and expirations. On applying the ear to the anterior surface of the chest, on the right side, a distinct sonorous ronchus was heard, if the child breathed with rapidity, or while crying, but when in a state of perfect repose, as while sleeping, not the slightest sound, even the natural murmur of respiration, could be heard. On the left side, the sound of respiration was at all times intense, except when masked by the sonorous ronchus above spoken of. It was observed too, that the left side of the chest, only expanded and contracted with respiration, and that the child could not lie long upon this side. These symptoms taken in connection with the history of the case, left no doubt of the existence of the foreign body in the air passages, and that this was situated in the right principal bronchial tube. An operation, being determined upon for its removal, was performed on the 19th of September, in the presence of Drs. Boone, Maxwell, and Blaney, in the following manner: An incision was made an inch and a half in length, directly in front of the larynx, and carried down to this organ by degrees, although the fits of suffocation, and a free venous hemorrhage which occurred, required a length of time to be consumed in this part of the operation. The crico-thyroid membrane was punctured, and the incision extended downward through the cricoid cartilage, and the two superior rings of the trachea, and upwards through a part of the thyroid cartilage. This produced another fit of coughing, so severe as to threaten suffocation, which was renewed as often as attempts were made to remove the foreign body, or to introduce a tube for the purpose of respiration. The orifice, therefore, being free, was left without a tube, measures suitable to combat inflammation were adopted, and directions given for the removal of the foreign body, should it present itself. This was favored by position, and as far as possible by other means, but without success, although it was noticed on the 20th, after a paroxysm of coughing, that the right side of the chest expanded during inspiration, the respiratory murmur was heard upon that side, indicating that the body had changed its situation. The respiration gradually became more frequent and difficult, and the child died on the 21st of Sept.

*Examination after Death.* A water melon seed was found in the trachea just at the point of its bifurcation, and considerable mucus was found in the bronchial tubes. The posterior three-fourths of the right lung, and half of the left, were found at a state of red hepatization, showing that an extensive pneumonia had been superadded to the bronchial affection; the two being sufficient, in all probability, to have proved fatal, even if the foreign body had been removed.

The dissection in this case, while it proved the urgent necessity of an operation, showed also, that it had been so long deferred, as to render its success doubtful if not impossible.

Chicago, October 1, 1845.

